# Supplementary material for: Increasing the Efficiency of Canola and Soybean GMO Detection and Quantification Using Multiplex Droplet Digital PCR
Source: Biology (Basel). 2022 Jan 27;11(2):201. doi: 10.3390/biology11020201 (PMC8869681; doi:10.3390/biology11020201)
Supplement: Supplementary file 1 [file biology-11-00201-s001.zip › biology-1537849-supplementary.pdf]

SUPPLEMENTARY MATERIALS

Increasing Efficiency of Canola and Soybean GMO Detection and Quantification with Multiplex Droplet Digital PCR

Tigst Demeke, Sung-Jong Lee and Monika Eng

Grain Research Laboratory, Canadian Grain Commission, Winnipeg, MB, R3C 3G8

Example of DNA mix preparation:

TETRAPLEX SAMPLE PREPARATION: Solutions of 20 ng/μL concentration were prepared for non-GM DNA and each GM event at 100%. These solutions were used to prepare the GM DNA mix as described below.

5% GM mix: 25 μL of each of the four GM solution at 100% was added to 400 μL non-GM DNA = 500 μL of tetraplex mix containing 5% of each GM event.

1% GM mix: 80 μL of the 5% GM mix (described above) was added to 320 μL of non-GM DNA = 400 μL of tetraplex containing 1% of each GM event.

0.1% GM mix: 35 μL of the 1% GM mix (described above) was added to 315 μL of non-GM DNA = 350 μL of tetraplex containing 0.1% of each GM event.

***duplex and triplex GM DNA mixes were prepared in the same manner using two and three GM events respectively

Supplementary Table S1. Primer and probe concentrations and probe labels used for duplex ddPCR assays

			Primer and probe concentrations and labels			
			Forward	Reverse	Probe	
Crop	Duplex events (μM)	Event		(μM)	(μM)	Label
Canola	HCN92 & GT73	HCN92	0.4	0.4	FAM	0.2
		GT73	0.4	0.4	HEX	0.2
	HCN92 & MON88302	HCN92	0.4	0.4	FAM	0.2
		MON88302	0.4	0.4	HEX	0.2
	GT73 & MON88302	GT73	0.4	0.4	FAM	0.2
		MON88302	0.4	0.4	HEX	0.2
	Reference gene	FatA(A)	0.3	0.9	HEX	0.15
Soybean	A2704 & DP305423	A2704	0.4	0.4	FAM	0.2
		DP305423	0.8	0.5	HEX	0.1
	A2704 & MON89788	A2704	0.4	0.4	FAM	0.2
		MON89788	0.4	0.4	HEX	0.2
	DP305423 & MON89788	DP305423	0.8	0.5	FAM	0.1
		MON89788	0.4	0.4	HEX	0.2
	A2704 & DAS81419	A2704	0.4	0.4	HEX	0.2
		DAS81419	0.4	0.4	FAM	0.2
	Reference gene	Lectin	0.4	0.4	FAM	0.2

Supplementary Table S2. Primer and probe concentrations and probe labels used for triplex ddPCR assays

			Primer and probe information			
			Forward	Reverse	Probe	
Crop	Triplex events	Event	(μM)	(μM)	Label	(μM)
Canola	HCN92, GT73	HCN92	0.4	0.4	FAM	0.4
	& MON88302	GT73	0.4	0.4	HEX	0.1
		MON88302	0.4	0.4	FAM/HEX	0.12
Canola	OXY235, GT73	OXY235	0.4	0.4	FAM	0.17
	& MON88302	GT73	0.4	0.4	HEX	0.17
		MON88302	0.4	0.4	FAM/HEX	0.13
Canola	Reference gene	FatA(A)	0.3	0.9	FAM	0.15
Soybean	A2704, MON89788	A2704	0.4	0.4	FAM	0.17
	& DP305423	MON89788	0.4	0.4	HEX	0.13
		DP305423	0.8	0.5	FAM/HEX	0.2
Soybean	MON89788, A2704	MON89788	0.4	0.4	FAM	0.2
	& DAS81419	A2704	0.4	0.4	HEX	0.2
		DAS81419	0.4	0.4	FAM/HEX	0.13
Soybean	Reference gene	Lectin	0.4	0.4	FAM	0.2

For the triplex ddPCR, MON88302 and DP305423 events were analyzed using a mixture of equal amounts of FAM and HEX labelled probes. Final concentration is the total amount of probe used.

Supplementary Table S3. Primer and probe concentrations and probe labels used for tetraplex ddPCR assays

			Primer and probe information			
			Forward	Reverse	Probe	
Crop	Tetraplex events	Event	(μM)	(μM)	Label	(μM)
Canola	HCN92, GT73,	HCN92	0.4	0.4	FAM	0.27
	MON88302, OXY235	GT73	0.4	0.4	HEX	0.30
		MON88302	0.4	0.4	FAM	0.30
		OXY235	0.4	0.4	HEX	0.17
	Reference gene	FatA(A)	0.3	0.9	HEX	0.15
Soybean	A2704, DP305423,	A2704	0.4	0.4	FAM	0.23
	MON89788 &	DP305423	0.8	0.5	HEX	0.17
	DAS81419	MON89788	0.4	0.4	FAM	0.10
		DAS81419	0.4	0.4	HEX	0.27
	Reference gene	Lectin	0.4	0.4	FAM	0.20
